# Effects of Dietary Supplementation with α-Mangostin on Oviduct Inflammation and Eggshell Quality in Aging Laying Hens

**DOI:** 10.3390/ani16071118

**Published:** 2026-04-05

**Authors:** Lu Huang, Ruixin Qin, Qianqian Yu, Qili Yan, Desheng Qi

**Affiliations:** School of Animal Science and Technology, Huazhong Agricultural University, Wuhan 430070, China

**Keywords:** α-mangostin, laying hen, eggshell quality, antioxidant, inflammation

## Abstract

This study investigated the effects of dietary supplementation with α-mangostin (α-Ma), a bioactive xanthone derived from mangosteen pericarp, on production performance and egg quality in late-phase laying hens. The experiment was conducted using a completely randomized design. In total, 576 healthy 51-week-old Beinong No. 2 laying hens were randomly assigned to 4 dietary treatments (*n* = 12): a basal diet (CON) or the basal diet supplemented with 80, 120, or 160 mg/kg α-Ma. The experiment lasted for 4 weeks, after which production performance, egg quality, serum biochemical and antioxidant parameters, inflammatory markers, and uterine gene expression were evaluated. Dietary supplementation with α-mangostin, particularly at 120 mg/kg, significantly improved feed efficiency (*p* < 0.05), as evidenced by a reduced feed-to-egg ratio from week 2 onward, without affecting average daily feed intake or egg production rate. After 4 weeks, hens receiving 120 mg/kg α-Ma exhibited significantly greater egg weight and eggshell strength (*p* < 0.05). Serum and hepatic antioxidant capacities were significantly enhanced, with increased glutathione peroxidase and catalase activities, elevated total antioxidant capacity, and decreased malondialdehyde levels (*p* < 0.05). Moreover, α-Ma at 120 mg/kg specifically lowered the concentration of the pro-inflammatory cytokine interleukin-1β in both serum and uterine tissue (*p* < 0.05). At the molecular level, this dosage significantly upregulated uterine genes essential for eggshell formation (*p* < 0.05), including calcium transporters (*TRPV6*, *ATP2B2*), the matrix protein gene *OC-116*, and other key genes (*LYZ*, *CA2*, *SLC4A9*, and *ATP6V0D2*). In conclusion, dietary supplementation with 120 mg/kg α-Ma effectively enhances feed efficiency, strengthens antioxidant and anti-inflammatory defenses, and upregulates uterine genes involved in biomineralization, thereby improving eggshell quality in aging laying hens. These findings support α-Ma as a promising plant-based feed additive for maintaining productivity and egg quality in antibiotic-free layer production systems.

## Simple Summary

As laying hens age, they often experience reduced eggshell quality and increased health problems due to inflammation and oxidative stress in their reproductive tissues. This study investigated whether adding α-mangostin (α-Ma) to the diet of 51-week-old laying hens could improve their oviduct inflammation and eggshell quality. The results showed that hens receiving 120 mg/kg of α-Ma for four weeks had better feed efficiency, stronger eggshells, and lower levels of inflammation and oxidative damage compared to hens that did not receive the supplement. It also enhanced the expression of genes responsible for calcium transport and eggshell formation. These findings suggest that α-Ma supplementation offers a natural and effective strategy to maintain hen health and egg quality during the late laying period, potentially supporting more sustainable and welfare-friendly egg production systems.

## 1. Introduction

Eggshell quality is a critical determinant of both the market value and economic profitability of table eggs. Current research on phytogenic feed additives in poultry has primarily focused on growth performance and intestinal health in broilers [1,2], with limited investigation into their direct effects on oviduct inflammation and eggshell quality in laying hens [3]. The global layer industry incurs billions of dollars in annual losses directly attributable to eggshell defects, with breakage rates rising sharply during the late laying period—often reaching 6% to 8% [4]. Issues such as thin-shelled, sandy-shelled, and soft-shelled eggs not only reduce farm profitability but also provide potential pathways for pathogenic microorganisms to contaminate eggs, posing serious threats to food safety [5]. Therefore, a thorough understanding of the underlying causes of eggshell quality defects and the development of effective mitigation strategies are essential for ensuring the sustainable growth of the poultry industry.

The decline in eggshell quality arises from multiple factors, with oviduct inflammation frequently identified as its core pathological basis. Aging in laying hens is associated with progressive structural and functional deterioration of the reproductive tract, including mitochondrial damage in ovarian tissues and increased cellular apoptosis in the uterus, which collectively impair calcium transport and eggshell formation [6,7]. Furthermore, aged hens exhibit reduced antioxidant capacity and heightened susceptibility to oxidative stress and inflammation, rendering them particularly vulnerable to oviductal inflammatory disorders that compromise both egg production and shell quality [3,8]. The oviduct—particularly the uterus (shell gland)—serves as the primary site for eggshell formation and calcification. Its proper function depends on a finely tuned balance involving calcium-transport proteins (such as CaBP-D28k), the carbonic anhydrase system, and local microbial homeostasis [9]. However, in commercial production, laying hens are continually exposed to various stressors. Pathogenic infections (e.g., by *Salmonella* or *Escherichia coli*) can activate the innate immune response in the oviduct mucosa, triggering a cascade of pro-inflammatory cytokines, including interleukin (IL)-1β and tumor necrosis factor-α (TNF-α), mainly through the Toll-like receptor 4/nuclear factor kappa-B (NF-κB) signaling pathway [10]. This inflammatory reaction damages mucosal epithelial cells and markedly suppresses the expression and activity of calcium-transport proteins, ultimately impairing calcium deposition [11]. In addition, nutritional and metabolic disturbances—such as vitamin D_3_ deficiency or an improper calcium-to-phosphorus ratio—as well as environmental heat stress, can provoke local inflammation and oxidative stress in the oviduct. These disruptions interfere with carbonic anhydrase activity and may induce respiratory alkalosis, disturbing the steady supply of carbonate and calcium ions essential for shell formation [12]. Collectively, these factors lead to abnormal synthesis of eggshell matrix proteins and disruption of the mineralization process, resulting in decreased shell strength and thickness.

Antibiotics were historically used extensively to control salpingitis; however, growing concerns over drug residues, bacterial resistance, and public health risks have driven the global poultry industry to pursue antibiotic-free alternatives. Phytogenic feed additives offer significant advantages in antibiotic-free production due to their antimicrobial and antioxidant properties that enhance gut health and reduce antibiotic dependence [13]. Currently, several substitutes—such as plant essential oils, probiotics, and chitosan—show promising potential, though each presents notable limitations [14]. For instance, high doses of essential oils can reduce feed palatability; probiotics often act too slowly to counter acute inflammation; and chitosan typically requires high inclusion levels to be effective, which limits its cost-efficiency [15,16]. Therefore, there is an urgent need to develop a natural, plant-derived additive with well-defined targets, strong efficacy, and high safety for the precise prevention and control of oviduct inflammation, ultimately improving eggshell quality.

In this context, α-mangostin (α-Ma), a xanthone compound derived from the pericarp of mangosteen (*Garcinia mangostana*), has attracted growing research interest [17]. A range of in vitro and in vivo studies suggest that α-Ma exerts multiple biological effects that may address the multifactorial nature of salpingitis. α-Mangostin exerts its anti-inflammatory effects primarily through inhibition of the NF-κB signaling pathway and modulation of calcium ion channels such as ORAI1 and TRPV3, thereby reducing the secretion of pro-inflammatory cytokines including IL-2 and IL-1β [18,19]. Given that inflammation in the shell gland suppresses the expression of calcium transporters (e.g., *TRPV6*, *ATP2B2*) and eggshell matrix proteins (e.g., *OC-116*) essential for proper biomineralization [9,20], the anti-inflammatory and calcium-regulating properties of α-mangostin may directly preserve or enhance the expression of these genes, thereby improving eggshell quality in aging laying hens. α-Ma exhibits strong antioxidant capacity, thus protecting oviduct tissues from oxidative damage [21]. It shows direct antibacterial effects against a variety of avian pathogens (e.g., *E. coli*), with a minimum inhibitory concentration significantly lower than that of many conventional essential oils [22]. Collectively, these properties suggest that α-Ma may help maintain oviduct health through multiple complementary mechanisms, offering a novel strategy to enhance eggshell quality.

Based on this background, Although previous research has demonstrated the anti-inflammatory and antioxidant properties of α-mangostin in various animal models, including poultry [1,23], most studies have focused on its general effects on growth performance or its application in broilers under disease challenge conditions [24,25]. To date, no comprehensive investigation has been conducted to evaluate the dose-dependent effects of dietary α-mangostin supplementation on oviduct health and eggshell quality in late-phase laying hens. The present study was performed to systematically evaluate the effects of dietary supplementation with different doses of α-Ma on production performance, eggshell quality, antioxidant capacity, inflammatory markers, and the expression of key functional genes in the oviduct of laying hens. We sought to clarify its underlying mechanisms of action and provide a robust scientific basis for the potential use of α-Ma as an effective phytogenic feed additive in layer production. Ultimately, the goal is to contribute to the prevention of salpingitis, promote animal welfare, and improve eggshell quality across the poultry industry.

## 2. Materials and Methods

### 2.1. Animals and Experimental Design

All experimental procedures involving animals were approved by the Animal Care and Use Committee of Huazhong Agricultural University. Informed consent was obtained from all animal owners involved in the study. In total, 576 healthy 51-week-old Beinong No. 2 laying hens were randomly assigned to 4 dietary treatment groups, each consisting of 12 replicates with 12 hens per replicate. The Beinong No. 2 is a commercial brown-egg layer breed developed in China, known for its high persistency of lay and excellent egg quality during the late production phase, making it a suitable model for studying age-related declines in eggshell quality. Hens were housed in 4-tiered battery cages, with 6 hens per cage (cage dimensions: 45 cm × 45 cm × 40 cm). Each cage was equipped with a trough feeder (45 cm in length), and two nipple drinkers providing ad libitum access to water. The experimental house was environmentally controlled with a temperature maintained at 22 ± 2 °C and a 16L:8D light cycle. The trial was conducted at a poultry farm of Hubei Rongjing Poultry Technology Development Co., Ltd. in Xiaogan City, Hubei Province, China. The temperature, humidity, and light intensity in the rearing environment were uniformly maintained and regulated by modern automated equipment. The trial lasted for 4 weeks, following a 1-week acclimation period. The 4-week experimental duration was selected based on previous studies [24,26].

### 2.2. Dietary Treatments

Four isonitrogenous and isocaloric experimental diets were formulated as follows: Control (CON): basal diet, MA-80: basal diet + 80 mg/kg α-Ma, MA-120: basal diet + 120 mg/kg α-Ma, and MA-160: basal diet + 160 mg/kg α-Ma. The α-Ma (purity ≥ 95%, confirmed by high-performance liquid chromatography) was purchased from Shaanxi Haochen Biotechnology Co., Ltd. (Xi’an, China) and thoroughly mixed into the basal diet. The basal diet was formulated to meet the nutrient requirements of laying hens according to the NRC (1994) guidelines [27] (Table 1). Feed and water were available ad libitum throughout the experimental period.

### 2.3. Data Collection and Sampling

Production Performance: Daily feed intake and the number of eggs produced were recorded per replicate on a weekly basis to calculate average daily feed intake, egg production rate, and feed-to-egg ratio (F/E). Feed-to-egg ratio (F/E, g feed/g egg) = total feed consumed per replicate (g)/total egg mass produced per replicate (g). The egg weight per replicate was also measured.

Egg Quality: For egg quality analysis at weeks 2 and 4, 30 eggs per treatment were randomly selected from the pooled collection of all eggs laid on the sampling day. This sample size (30 eggs per treatment) is consistent with standard practices based on previous studies [28,29]. From each individual egg, egg weight, eggshell strength, eggshell thickness, albumen height, Haugh unit, and yolk color were determined using an egg quality analyzer (Fujihira Industry Co., Ltd., Tokyo, Japan).

Serum and Tissue Sampling: At the conclusion of the 4-week trial, one hen from each replicate (12 hens per treatment) was randomly selected after a 12 h fasting period. Blood samples were collected from the wing vein, centrifuged at 3000× *g* for 15 min to obtain serum, and stored at −80 °C. Following exsanguination, the liver and the uterine segment of the oviduct were immediately dissected. Portions of each tissue were snap-frozen in liquid nitrogen and stored at −80 °C for subsequent RNA and biochemical analyses.

### 2.4. Biochemical Analyses

Serum biochemical parameters, such as alanine aminotransferase, aspartate aminotransferase, total protein, albumin, triglycerides, total cholesterol, and alkaline phosphatase, were measured using an automatic biochemistry analyzer (Hitachi 7100, Hitachi High-Technologies Corporation, Tokyo, Japan) along with the appropriate commercial assay kits.

Tissue homogenate preparation: Frozen uterine tissue samples were thawed on ice and approximately 100 mg of tissue was accurately weighed. The tissue was minced with scissors and homogenized in ice-cold phosphate-buffered saline (PBS, 0.01 M, pH 7.4) at a ratio of 1:9 (*w*/*v*) using an IKA T10 basic Ultra-Turrax homogenizer (IKA Works, Staufen, Germany) at 20,000 rpm for 30–60 s on ice. The homogenate was centrifuged at 12,000× *g* for 15 min at 4 °C, and the supernatant was collected and stored at −80 °C until analysis. Total protein concentration in the supernatant was determined using a bicinchoninic acid (BCA) protein assay kit (Pierce, Rockford, IL, USA) with bovine serum albumin as standard.

The serum and liver homogenates’ antioxidant indices—such as total antioxidant capacity (T-AOC), glutathione peroxidase (GSH-Px) activity, catalase (CAT) activity, and malondialdehyde (MDA) concentration—were assessed utilizing colorimetric commercial kits (Nanjing Jiancheng Bioengineering Institute, Nanjing, China) in accordance with the manufacturer’s guidelines.

Concentrations of inflammatory cytokines (IL-1β, IL-6, IL-10, and TNF-α) in serum and uterine tissue homogenates were quantified using chicken-specific enzyme-linked immunosorbent assay kits (Cusabio Technology LLC, Wuhan, China) following the manufacturer’s protocols.

### 2.5. Real-Time Polymerase Chain Reaction Analysis

Total RNA was extracted from uterine tissue using TRIzol reagent (Invitrogen, Carlsbad, CA, USA), and RNA quality and concentration were assessed spectrophotometrically. First-strand cDNA was synthesized using the PrimeScript RT reagent kit (Takara, Shiga, Japan). Real-time polymerase chain reaction was performed on a QuantStudio 5 system (Applied Biosystems, Waltham, MA, USA) using SYBR Green Premix (Takara, Shiga, Japan). Relative mRNA expression levels of target genes (*OC-116*, *OC-17*, *TRPV6*, *ATP2B2*, *LYZ*, *EDIL3*, *CA2*, *SLC4A1*, *SLC4A2*, *SLC4A7*, *SLC4A9*, *ATP6V0D2*, and *ATP6V1G3*) were calculated using the 2^−ΔΔCt^ method, with GAPDH serving as reference genes. The primer sequences are listed in Supplementary Table S1.

### 2.6. Statistical Analysis

All data were analyzed using one-way analysis of variance in the GLM procedure of SAS 9.4 (SAS Institute Inc., Cary, NC, USA). Differences among treatment means were determined using Duncan’s multiple range test. Results are expressed as means with pooled standard error of the mean, and statistical significance was declared at *p* < 0.05.

## 3. Results

### 3.1. Effects of α-Ma on Production Performance in Laying Hens

Effects of α-Ma on Production Performance in Laying Hens are presented in Table 2. Dietary supplementation with α-Ma had no significant effect (*p* > 0.05) on the average daily feed intake or egg production rate of laying hens compared with the control group. After 1 week of feeding, the 120 mg/kg supplementation group showed a significantly lower F/E than the control group (*p* < 0.05). By week 2, all three α-Ma supplementation groups exhibited significantly reduced F/E values (*p* < 0.05). At week 3, all α-Ma groups continued to maintain significantly lower F/E values, with the 120 mg/kg group showing the most pronounced improvement—significantly lower than both the 80 mg/kg and 160 mg/kg groups (*p* < 0.05). After 4 weeks, the 80 mg/kg and 120 mg/kg groups still exhibited significantly reduced F/E, and the reduction in the 120 mg/kg group remained significantly greater than that in the 80 mg/kg group (*p* < 0.05).

### 3.2. Effects of α-Ma on Egg Quality

Effects of α-Ma on Egg Quality are summarized in Table 3. After 2 weeks of feeding, none of the α-Ma supplementation groups showed significant differences (*p* > 0.05) in egg weight, eggshell strength, eggshell thickness, albumen height, yolk color, or Haugh unit compared with the control group. However, after 4 weeks, the 120 mg/kg α-Ma group exhibited significantly higher egg weight and eggshell strength (*p* < 0.05). In addition, after 4 weeks, all three α-Ma supplementation groups showed a significant reduction in yolk color (*p* < 0.05).

### 3.3. Effects of α-Ma on Serum Biochemical Parameters in Laying Hens

As shown in Table 4, the 160 mg/kg α-Ma group showed a significantly higher serum ALB concentration than the control group (*p* < 0.05). No significant differences (*p* > 0.05) were observed among the groups for serum alanine aminotransferase, aspartate aminotransferase, triglycerides, total cholesterol, alkaline phosphatase, or total protein.

### 3.4. Effects of α-Ma on Serum Antioxidant Capacity in Laying Hens

Dietary supplementation with α-Ma significantly increased serum GSH-Px and CAT activities and significantly decreased MDA levels compared with the control group (*p* < 0.05) (Table 5). Additionally, supplementation with 80 mg/kg and 160 mg/kg α-Ma significantly enhanced T-AOC in serum (*p* < 0.05) (Table 5).

### 3.5. Effects of α-Ma on Hepatic Antioxidant Capacity in Laying Hens

Compared with the control group, dietary α-Ma supplementation significantly increased hepatic CAT activity and decreased the hepatic MDA concentration (*p* < 0.05) (Table 6). Hepatic T-AOC was also significantly improved in the 80 mg/kg α-Ma groups (*p* < 0.05) (Table 6). Furthermore, supplementation with 160 mg/kg α-Ma significantly enhanced hepatic GSH-Px activity (*p* < 0.05) (Table 6).

### 3.6. Effects of α-Ma on Serum Inflammatory Cytokines in Laying Hens

Effects of α-Ma on Serum Inflammatory Cytokines in Laying Hens are presented in Table 7. Dietary supplementation with 120 mg/kg α-Ma significantly reduced the serum IL-1β concentration compared with the control group (*p* < 0.05). No significant differences in the IL-10, IL-6, or TNF-α concentrations were observed among the groups (*p* > 0.05).

### 3.7. Effects of α-Ma on Inflammatory Cytokines in the Uterus of Laying Hens

Dietary supplementation with 120 mg/kg α-Ma significantly decreased the concentration of IL-1β in uterine tissue compared with the control group (*p* < 0.05) (Table 8). The levels of IL-10, IL-6, and TNF-α in the uterus were not significantly affected by any of the α-Ma supplementation levels (*p* > 0.05) (Table 8).

### 3.8. Effects of α-Ma on the Expression of Eggshell Formation-Related Genes

#### 3.8.1. Effects on Eggshell-Specific Matrix Protein Gene Expression

**Figure 1:**
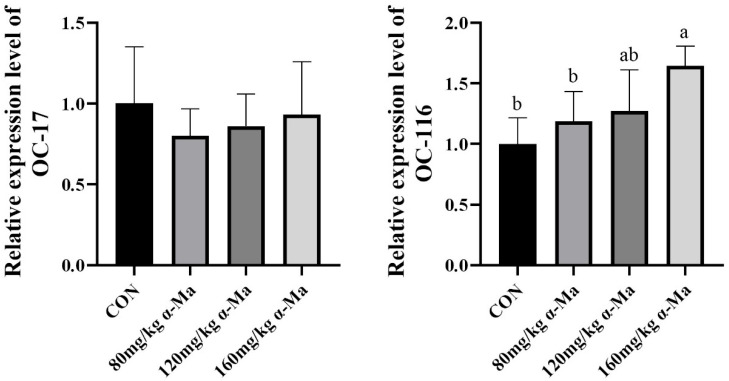
Effects on Eggshell-Specific Matrix Protein Gene Expression. Labeled means in a row without a common superscript letter differ, *p* < 0.05.

Dietary supplementation with 120 mg/kg α-Ma significantly up-regulated the expression of *OC-116* compared with the control group (*p* < 0.05) (Figure 1). The expression of *OC-17* was not significantly affected by any of the α-Ma doses.

#### 3.8.2. Effects on Genes Related to Calcium Ion (Ca^2+^) Transport in the Uterus

**Figure 2:**
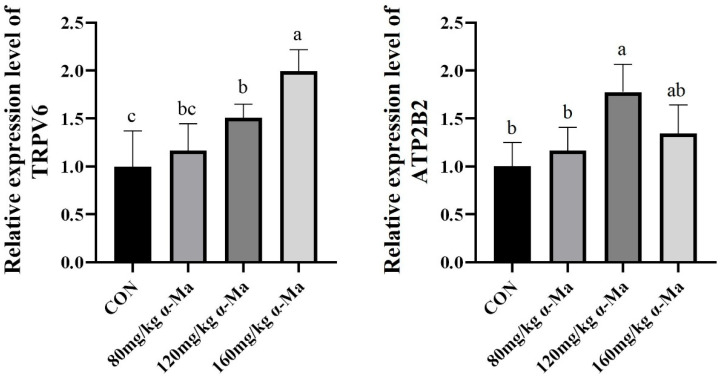
Effects on Genes Related to Calcium Ion (Ca^2+^) Transport in the Uterus. Labeled means in a row without a common superscript letter differ, *p* < 0.05.

Supplementation with 120 mg/kg and 160 mg/kg α-Ma significantly up-regulated the expression of *TRPV6* compared with the control (*p* < 0.05) (Figure 2). The 120 mg/kg group also showed a significant increase in *ATP2B2* expression (*p* < 0.05) (Figure 2).

#### 3.8.3. Effects on Genes Related to Calcium Carbonate Transport in the Uterus

**Figure 3:**
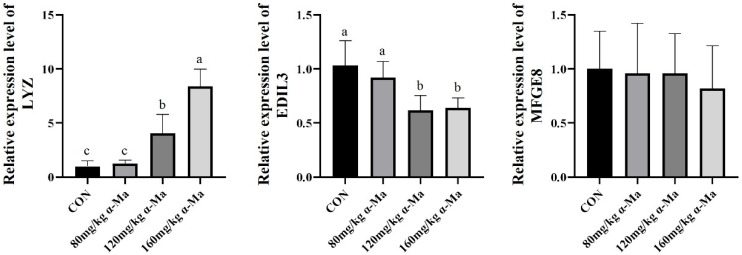
Effects on Genes Related to Calcium Carbonate Transport in the Uterus. Labeled means in a row without a common superscript letter differ, *p* < 0.05.

Compared with the control group, dietary supplementation with 120 mg/kg and 160 mg/kg α-Ma significantly up-regulated *LYZ* expression and significantly down-regulated *EDIL3* expression (*p* < 0.05) (Figure 3).

#### 3.8.4. Effects on Genes Related to Bicarbonate Transport in the Uterus

**Figure 4:**
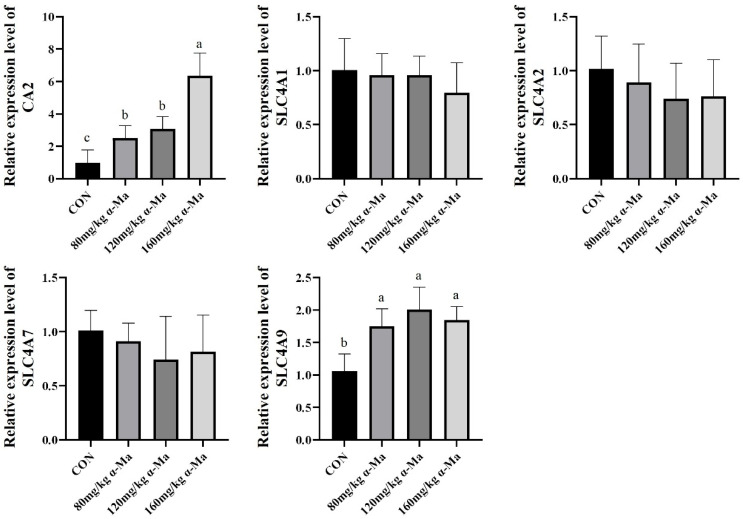
Effects on Genes Related to Bicarbonate Transport in the Uterus. Labeled means in a row without a common superscript letter differ, *p* < 0.05.

Dietary α-Ma supplementation significantly increased the expression of *CA2* and *SLC4A9* compared with the control group (*p* < 0.05) (Figure 4). No significant differences were observed among the groups for *SLC4A1*, *SLC4A2*, or *SLC4A7* expression.

#### 3.8.5. Effects on Genes Related to Proton Transport in the Uterus

**Figure 5:**
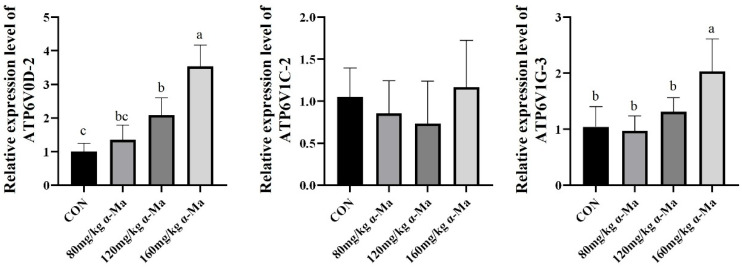
Effects on Genes Related to Proton Transport in the Uterus. Labeled means in a row without a common superscript letter differ, *p* < 0.05.

Supplementation with 120 mg/kg and 160 mg/kg α-Ma significantly up-regulated the expression of *ATP6V0D2* compared with the control (*p* < 0.05) (Figure 5). The 160 mg/kg group also showed a significant increase in *ATP6V1G3* expression (*p* < 0.05) (Figure 5).

## 4. Discussion

This study systematically evaluated the effects of dietary supplementation with different doses of α-Ma on production performance, health status, and eggshell quality in laying hens. The results showed that α-Ma significantly improved feed efficiency, enhanced systemic antioxidant capacity, alleviated both local and systemic inflammation, and increased eggshell strength, with these effects exhibiting clear dose- and time-dependent patterns. By comparing the present findings with previous studies, this discussion seeks to elucidate the multifaceted mechanisms underlying the action of α-Ma, and explore the potential factors that may account for these differences.

The improvement in production performance induced by α-Ma was primarily reflected in enhanced feed utilization efficiency. The F/E serves as a key economic indicator of how effectively feed is converted into eggs in laying hens. The values observed in our control group (2.3–2.6 g feed/g egg) are within the expected range for 51–55 week old laying hens, particularly under commercial production conditions [30,31]. This age-related increase in FCR is attributable to several physiological factors, including increased body weight leading to higher maintenance energy requirements, declining metabolic efficiency, and slightly reduced egg output during the late laying period [32,33]. In this study, α-Ma supplementation—particularly at 120 mg/kg—significantly reduced the F/E, and this effect became more pronounced with longer feeding duration, without adversely affecting feed intake or egg production rate. This finding is consistent with Jianfei Zhu et al., who reported that an appropriate dose of α-Ma improved the feed conversion rate in layers by enhancing nutrient digestibility and utilization without impairing normal production [34]. The underlying mechanisms may involve two main aspects: first, maintaining intestinal microecological balance through α-Ma’s antibacterial activity [22]; and second, strengthening intestinal barrier function by upregulating the expression of gut defense–related genes such as *BPIFB3* [35], thereby facilitating nutrient absorption. Notably, the 160 mg/kg supplementation group exhibited a weaker improvement in F/E compared with the 120 mg/kg group. Combined with the significantly elevated serum ALB concentration observed in this group, this suggests that the higher dose may have induced mild metabolic stress, approaching the metabolic tolerance threshold of the laying hens.

In terms of antioxidant function, α-Ma exhibited strong in vivo antioxidant activity. Oxidative stress is a major contributor to cellular damage and functional decline in animals. In this study, all α-Ma supplementation groups significantly increased serum GSH-Px and CAT activities while reducing MDA levels, a key marker of lipid peroxidation. In hepatic tissue, α-Ma at 80 and 160 mg/kg significantly enhanced T-AOC. These findings indicate that α-Ma effectively strengthens both systemic and hepatic antioxidant defenses, consistent with the observations of Tianhua Fu et al. (2018), who reported the potent free radical-scavenging ability of α-Ma in mammalian models [36]. The antioxidant effects of α-Ma are attributed to two complementary mechanisms: the direct scavenging of reactive oxygen species via its xanthone structure, and the activation of endogenous antioxidant response elements through its role as an exogenous signaling molecule [37]. The xanthone structure of α-mangostin possesses potent radical-scavenging properties that directly neutralize reactive oxygen species (ROS), thereby reducing oxidative damage to cellular membranes and lowering MDA formation [38,39]. In addition, α-mangostin may function as an exogenous signaling molecule that activates the nuclear factor erythroid 2-related factor 2 (Nrf2) pathway, a master regulator of antioxidant defense. Upon activation, Nrf2 translocates to the nucleus and binds to antioxidant response elements (ARE), upregulating the transcription of phase II detoxifying enzymes including GSH-Px and CAT [40]. This dual mechanism—direct radical scavenging coupled with indirect enhancement of endogenous antioxidant enzyme systems—explains the coordinated increase in both GSH-Px and CAT activities observed in the present study.

In this study, supplementation with 120 mg/kg α-Ma specifically and significantly reduced IL-1β concentrations in both serum and uterine tissue, while having no significant influence on other inflammatory cytokines such as IL-6, TNF-α, or IL-10.

The selective suppression of IL-1β while other cytokines remained unaffected can be explained by the specific mechanism of α-Ma as an NF-κB pathway inhibitor. α-Mangostin has been demonstrated to inhibit the translocation of NF-κB to the nucleus by preventing IκBα phosphorylation and degradation, thereby reducing the transcription of NF-κB target genes [41]. Importantly, IL-1β is not only a downstream target of NF-κB activation but also functions as an upstream activator of the same pathway [42]. While some studies have reported that α-Ma suppresses multiple pro-inflammatory cytokines including TNF-α and IL-6 [17,43], others have demonstrated that the inhibitory effects can vary depending on cell type, inflammatory stimulus, and dosage [44]. For instance, Mohan et al. (2018) found that α-Ma significantly inhibited TNF-α and IL-1β in peritoneal fluids, but the magnitude of suppression differed between these cytokines [45]. The selective IL-1β suppression observed in our study may reflect the specific physiological conditions of aging laying hens, where chronic low-grade inflammation differs from acute inflammatory models. Other cytokines such as IL-6 and TNF-α, which are regulated by additional signaling pathways (including MAPK and AP-1) that could remain partially active under the 120 mg/kg dose used in our study [46]. The dose-dependent nature of α-Ma’s effects further supports this interpretation, as higher concentrations may be required to achieve broader cytokine suppression [41]. The reduction in local inflammation in the oviduct is particularly important because it helps maintain the stable microenvironment necessary for proper eggshell formation.

The improvement in eggshell strength induced by α-Ma represents a clear manifestation of its multi-target actions. A significant increase in eggshell strength was observed in the 120 mg/kg α-Ma group after 4 weeks of feeding. Eggshell strength is largely determined by its ultrastructure (e.g., mammillary layer density) and chemical composition, and any factor that enhances the calcification process or the synthesis of matrix proteins can contribute to improved shell integrity [47]. At the molecular level, our study revealed that 120 mg/kg α-Ma significantly upregulated the expression of uterine calcium transporter genes (*TRPV6*, *ATP2B2*) and the eggshell matrix protein gene *OC-116*. As established by Jonchère et al. (2012), TRPV6 functions as the primary channel for basolateral calcium uptake from the bloodstream into uterine epithelial cells, while ATP2B2 mediates apical calcium extrusion into the uterine lumen where eggshell mineralization occurs [48,49]. The coordinated upregulation of these transporters increases the availability of ionic calcium (Ca^2+^) at the mineralization front, providing more substrate for calcium carbonate (CaCO_3_) crystal formation during the active calcification phase. Functional studies have demonstrated that OC-116 plays a critical role in regulating calcite crystal growth and morphology during eggshell calcification [50]. The protein interacts with the growing crystal surface, modulating crystal size and orientation to produce the characteristic columnar structure of the palisade layer, which is essential for mechanical strength [51]. Furthermore, the downregulation of EDIL3 (EGF-like repeats and discoidin I-like domains 3) in the 120 mg/kg and 160 mg/kg groups may contribute to optimized mineralization. EDIL3 has been identified as a major calcium-binding protein in the eggshell matrix, and its reduced expression during active calcification may facilitate proper crystal nucleation and growth by modulating the local ionic environment [52]. TRPV6 mediates Ca^2+^ influx across the cell membrane, ATP2B2 pumps Ca^2+^ out of the cell into the uterine lumen, and ovocleidin-116 (encoded by *OC-116*) is a critical matrix protein that regulates calcium crystal growth [20]. These gene expression changes form an integrated mechanism: enhanced TRPV6 and ATP2B2 expression increases Ca^2+^ supply efficiency, while upregulated OC-116 optimizes crystal growth regulation and matrix organization. The combination of increased substrate availability and improved structural guidance results in a denser, more organized palisade layer with reduced mammillary layer spacing—ultimately translating to the significant increase in eggshell strength observed in the 120 mg/kg group.

The findings of this study align with current understanding of the relationship between eggshell quality and the physiological status of laying hens. By demonstrating that dietary supplementation with 120 mg/kg α-Ma effectively improves feed efficiency, enhances antioxidant status, selectively suppresses oviduct inflammation, and upregulates key genes involved in calcium transport and eggshell matrix formation, our research provides a scientifically validated nutritional strategy for addressing the age-related decline in eggshell quality that plagues late-phase production. This is particularly relevant as producers worldwide seek effective antibiotic-free solutions to extend the productive lifespan of laying flocks while maintaining egg quality and hen welfare [53,54]. The significant improvement in eggshell strength observed with 120 mg/kg α-Ma supplementation directly translates to reduced egg breakage during collection, packaging, and transportation—a key factor influencing economic returns for producers [4,55]. Additionally, the enhancement in feed efficiency offers substantial cost savings in commercial operations, where feed represents the largest production expense [56]. Furthermore, by mitigating oxidative stress and inflammation in aging hens, α-Ma supplementation may contribute to improved animal welfare and reduced mortality during the extended production cycle [8]. Interestingly, all α-Ma supplementation groups produced significantly paler yolk color. Whether this effect arises from α-Ma or its metabolites competitively binding to lipid transport carriers—thereby interfering with carotenoid deposition in the yolk—remains to be clarified through further investigation. Furthermore, the 4-week experimental duration, while sufficient to detect significant improvements in production performance and eggshell quality, does not provide information on the long-term efficacy or sustainability of α-Ma supplementation. Future studies should extend the feeding period to 8 weeks or longer to evaluate whether the observed benefits are maintained or enhanced throughout the late production phase. Although we identified significant changes in the expression of genes critical for eggshell formation, the precise molecular mechanisms by which α-mangostin regulates these genes remain to be fully elucidated.

Overall, this study demonstrates that dietary supplementation with 120 mg/kg α-Ma effectively improves F/E, health status, and eggshell strength in late-phase laying hens by enhancing boosting systemic antioxidant capacity, selectively inhibiting the key pro-inflammatory cytokine IL-1β, and synergistically upregulating the expression of calcium transport and matrix protein genes in the uterus. These findings provide a solid theoretical basis for the use of α-Ma as a multi-target phytogenic feed additive in the sustainable and healthy production of laying hens.

## 5. Conclusions

Based on the findings of this study, dietary supplementation with α-Ma—particularly at 120 mg/kg—can be recommended as a practical nutritional strategy to enhance productivity and egg quality in late-phase laying hens. This optimal dosage significantly improved feed efficiency without adversely affecting egg production, accompanied by marked enhancement of antioxidant capacity and selective reduction in systemic and uterine inflammation. These physiological improvements translated into a clear increase in eggshell quality, primarily through the upregulation of key genes involved in calcium transport and matrix protein formation within the shell gland. Overall, α-Ma represents an effective, plant-derived additive that supports sustainable egg production by mitigating age-related declines in eggshell quality and maintaining the overall health of laying hens.

## Figures and Tables

**Table 1:** Ingredient composition and calculated nutrient levels of the basal diet (as-fed basis).

Ingredients	Content (%)	Calculated Nutrient Levels	Content
Corn	64.00	Metabolizable energy (ME, kcal/kg)	2750
Soybean meal (44% CP)	24.00	Crude protein (%)	16.50
Limestone	8.50	Calcium (%)	3.60
Dicalcium phosphate	1.50	Available phosphorus (%)	0.42
Soybean oil	1.00	Lysine (%)	0.85
Salt	0.30	Methionine (%)	0.42
DL-Methionine	0.15	Methionine + Cysteine (%)	0.72
L-Lysine HCl	0.05	Threonine (%)	0.62
Vitamin-mineral premix ^1^	0.50	Tryptophan (%)	0.18
Total	100.00	Linoleic acid (%)	1.20

^1^ The vitamin-mineral premix provided per kg of diet: vitamin A, 10,000 IU; vitamin D_3_, 3000 IU; vitamin E, 30 IU; vitamin K_3_, 2 mg; vitamin B_1_, 1.5 mg; vitamin B_2_, 6 mg; vitamin B_6_, 3 mg; vitamin B_12_, 0.02 mg; niacin, 30 mg; pantothenic acid, 15 mg; folic acid, 1 mg; biotin, 0.1 mg; choline chloride, 500 mg; Fe (as FeSO_4_·H_2_O), 60 mg; Cu (as CuSO_4_·5H_2_O), 8 mg; Mn (as MnSO_4_·H_2_O), 80 mg; Zn (as ZnSO_4_·H_2_O), 60 mg; I (as KI), 0.7 mg; Se (as Na_2_SeO_3_), 0.3 mg.

**Table 2:** Effects of α-Ma on Production Performance in Laying Hens.

Items	CON	80 mg/kg α-Ma	120 mg/kg α-Ma	160 mg/kg α-Ma	*p* Value
Week 1					
ADFI, g/d	94.32 ± 9.32	101.79 ± 11.23	99.77 ± 5.63	95.33 ± 11.45	0.19
Laying rate, %	79.51 ± 4.52	79.78 ± 2.90	82.43 ± 2.29	79.35 ± 4.01	0.41
Egg weight, g/egg	50.45 ± 3.26	53.50 ± 3.35	53.76 ± 2.10	52.06 ± 3.67	0.62
F/E	2.36 ± 0.09 ^a^	2.39 ± 0.07 ^a^	2.25 ± 0.14 ^b^	2.31 ± 0.08 ^a^	<0.001
Week 2					
ADFI, g/d	91.74 ± 7.60	92.62 ± 8.36	94.95 ± 8.63	93.25 ± 8.67	0.81
Laying rate, %	79.41 ± 1.98	80.52 ± 3.42	78.27 ± 2.24	80.25 ± 1.57	0.47
Egg weight, g/egg	50.06 ± 2.45	52.30 ± 2.75	55.38 ± 2.39	52.37 ± 2.61	0.08
F/E	2.31 ± 0.10 ^a^	2.20 ± 0.12 ^b^	2.19 ± 0.07 ^b^	2.22 ± 0.06 ^b^	0.003
Week 3					
ADFI, g/d	90.16 ± 7.99	91.10 ± 10.05	92.36 ± 7.04	89.91 ± 11.39	0.92
Laying rate, %	63.68 ± 4.87	67.37 ± 4.41	67.97 ± 4.17	66.54 ± 5.87	0.38
Egg weight, g/egg	50.93 ± 3.15	52.38 ± 3.45	55.76 ± 2.50	52.16 ± 3.82	0.21
F/E	2.79 ± 0.13 ^a^	2.58 ± 0.12 ^b^	2.43 ± 0.07 ^c^	2.59 ± 0.09 ^b^	<0.001
Week 4					
ADFI, g/d	93.16 ± 9.51	92.53 ± 11.31	92.90 ± 8.98	92.82 ± 6.71	0.99
Laying rate, %	70.61 ± 3.97	70.74 ± 4.58	69.19 ± 7.63	69.59 ± 7.02	0.23
Egg weight, g/egg	50.37 ± 3.04	52.13 ± 3.50	55.49 ± 2.91	52.58 ± 2.74	0.12
F/E	2.62 ± 0.09 ^a^	2.51 ± 0.08 ^b^	2.42 ± 0.06 ^c^	2.54 ± 0.06 ^a^	<0.001

Values are means ± SE, *n* = 12. Labeled means in a row without a common superscript letter differ, *p* < 0.05. ADFI: Average daily feed intake; F/E: g feed/g egg.

**Table 3:** Effects of α-Ma on Egg Quality.

Items	CON	80 mg/kg α-Ma	120 mg/kg α-Ma	160 mg/kg α-Ma	*p* Value
Week 2					
Weight, g/egg	52.85 ± 3.66	52.24 ± 3.28	54.34 ± 3.09	54.35 ± 3.65	0.33
Shell breaking strength, N	33.77 ± 9.74	37.98 ± 10.08	38.64 ± 9.66	35.97 ± 6.67	0.37
Shell thickness, mm	0.32 ± 0.01	0.32 ± 0.01	0.33 ± 0.01	0.33 ± 0.01	0.89
Albumen height, mm	5.88 ± 1.83	5.34 ± 0.94	5.78 ± 0.59	6.35 ± 1.33	0.29
Yolk color	5.62 ± 0.57	6.26 ± 0.46	6.19 ± 0.43	6.01 ± 0.63	0.05
Haugh unit	76.84 ± 13.66	74.42 ± 8.10	77.11 ± 4.19	80.33 ± 10.38	0.53
Week 4					
Weight, g/egg	49.66 ± 5.11 ^b^	49.39 ± 2.99 ^b^	55.22 ± 4.63 ^a^	51.60 ± 3.41 ^ab^	0.004
Shell breaking strength, N	28.38 ± 7.97 ^b^	36.54 ± 10.41 ^ab^	38.33 ± 9.34 ^a^	34.53 ± 8.89 ^ab^	0.04
Shell thickness, mm	0.32 ± 0.01	0.32 ± 0.01	0.33 ± 0.01	0.32 ± 0.01	0.87
Albumen height, mm	5.45 ± 0.78	5.37 ± 0.89	5.13 ± 0.79	5.56 ± 1.23	0.74
Yolk color	7.83 ± 0.55 ^a^	5.93 ± 1.08 ^b^	5.99 ± 0.81 ^b^	5.89 ± 0.78 ^b^	<0.001
Haugh unit	75.53 ± 5.39	75.92 ± 6.03	71.48 ± 5.95	75.85 ± 8.02	0.31

Values are means ± SE, *n* = 30. Labeled means in a row without a common superscript letter differ, *p* < 0.05.

**Table 4:** Effects of α-Ma on Serum Biochemical Parameters in Laying Hens.

Items	CON	80 mg/kg α-Ma	120 mg/kg α-Ma	160 mg/kg α-Ma	*p* Value
ALB (g/L)	15.11 ± 1.76 ^b^	16.58 ± 2.04 ^ab^	16.52 ± 1.52 ^ab^	17.00 ± 2.29 ^a^	0.04
ALT (U/L)	16.58 ± 10.11	14.74 ± 4.07	21.63 ± 11.70	21.79 ± 15.46	0.52
AST (U/L)	227.24 ± 38.92	243.94 ± 83.81	242.72 ± 40.44	242.33 ± 43.29	0.77
TG (mmol/L)	15.74 ± 5.88	12.65 ± 2.41	15.51 ± 4.17	13.73 ± 4.77	0.42
TC (mmol/L)	3.63 ± 1.52	3.23 ± 1.20	3.91 ± 1.40	3.14 ± 0.93	0.45
ALP (U/L)	253.83 ± 106.13	331.08 ± 96.62	343.72 ± 155.09	332.55 ± 142.14	0.31
TP (g/L)	48.93 ± 6.13	53.88 ± 9.89	51.36 ± 7.29	58.67 ± 17.17	0.18

Values are means ± SE, *n* = 12. Labeled means in a row without a common superscript letter differ, *p* < 0.05. ALB: Albumin; ALT: Alanine aminotransferase; AST: Aspartate aminotransferase; TG: triglyceride; TC: total cholesterol; ALP: alkaline phosphatase; TP: Total Protein.

**Table 5:** Effects of α-Ma on Serum Antioxidant Capacity in Laying Hens.

Items	CON	80 mg/kg α-Ma	120 mg/kg α-Ma	160 mg/kg α-Ma	*p* Value
T-AOC (U/mL)	8.43 ± 1.26 ^b^	12.05 ± 1.22 ^a^	10.54 ± 1.39 ^ab^	11.21 ± 1.17 ^a^	<0.01
GSH-Px (U/mL)	1020.62 ± 40.82 ^b^	1561.55 ± 61.24 ^a^	1326.81 ± 86.17 ^a^	1388.04 ± 34.66 ^a^	<0.001
CAT (U/mL)	9.24 ± 3.23 ^b^	16.17 ± 2.99 ^a^	13.21 ± 2.74 ^a^	13.49 ± 2.81 ^a^	<0.01
MDA (nmol/mL)	4.87 ± 1.17 ^a^	2.00 ± 0.84 ^b^	2.39 ± 0.31 ^b^	1.89 ± 0.83 ^b^	<0.01

Values are means ± SE, *n* = 12. Labeled means in a row without a common superscript letter differ, *p* < 0.05.

**Table 6:** Effects of α-Ma on Hepatic Antioxidant Capacity in Laying Hens.

Items	CON	80 mg/kg α-Ma	120 mg/kg α-Ma	160 mg/kg α-Ma	*p* Value
T-AOC (U/mg prot)	2.98 ± 0.69 ^b^	4.35 ± 0.41 ^a^	3.31 ± 0.48 ^b^	3.73 ± 0.46 ^ab^	0.01
GSH-Px (U/mg prot)	29.17 ± 6.13 ^b^	30.92 ± 7.42 ^b^	31.50 ± 7.11 ^b^	40.25 ± 7.02 ^a^	0.03
CAT (U/mg prot)	62.06 ± 19.86 ^b^	133.43 ± 17.42 ^a^	111.71 ± 18.37 ^a^	123.50 ± 24.22 ^a^	<0.01
MDA (nmol/mg prot)	0.58 ± 0.14 ^a^	0.15 ± 0.08 ^b^	0.18 ± 0.07 ^b^	0.17 ± 0.09 ^b^	<0.001

Values are means ± SE, *n* = 12. Labeled means in a row without a common superscript letter differ, *p* < 0.05.

**Table 7:** Effects of α-Ma on Serum Inflammatory Cytokines in Laying Hens.

Items	CON	80 mg/kg α-Ma	120 mg/kg α-Ma	160 mg/kg α-Ma	*p* Value
IL-1β (pg/mL)	24.36 ± 2.68 ^a^	18.51 ± 5.73 ^ab^	14.37 ± 4.10 ^b^	17.30 ± 3.26 ^b^	0.03
IL-6 (pg/mL)	46.11 ± 12.91	53.03 ± 7.38	48.88 ± 7.95	50.26 ± 6.79	0.53
TNF-α (pg/mL)	84.16 ± 6.73	83.32 ± 9.04	84.11 ± 8.17	84.34 ± 6.11	0.98
IL-10 (pg/mL)	9.19 ± 1.65	9.65 ± 1.01	9.83 ± 1.06	10.11 ± 1.17	0.59

Values are means ± SE, *n* = 12. Labeled means in a row without a common superscript letter differ, *p* < 0.05.

**Table 8:** Effects of α-Ma on Inflammatory Cytokines in the Uterus of Laying Hens.

Items	CON	80 mg/kg α-Ma	120 mg/kg α-Ma	160 mg/kg α-Ma	*p* Value
IL-1β (pg/mg prot)	16.84 ± 3.70 ^a^	12.97 ± 3.84 ^ab^	11.96 ± 1.52 ^b^	13.81 ± 2.02 ^ab^	0.04
IL-6 (pg/mg prot)	13.54 ± 1.22	13.28 ± 2.84	13.49 ± 2.75	13.58 ± 2.08	0.99
TNF-α (pg/mg prot)	10.73 ± 3.01	9.76 ± 2.25	10.30 ± 1.61	10.52 ± 1.83	0.82
IL-10 (pg/mg prot)	15.88 ± 2.24	17.15 ± 2.86	17.17 ± 4.29	19.29 ± 4.56	0.28

Values are means ± SE, *n* = 12. Labeled means in a row without a common superscript letter differ, *p* < 0.05.

## Data Availability

The data that support the findings of this study are available from the corresponding author upon reasonable request.

## Supplementary Materials

The following supporting information can be downloaded at: https://www.mdpi.com/article/10.3390/ani16071118/s1, Table S1: Primer sequences for PCR reactions.

## References

[1] Sriboonyong P., Poommarin P., Sittiya J., Opanasopit P., Ngawhirunpat T., Patrojanasophon P., Pornpitchanarong C. (2022). The utilization of mangosteen pericarp extract for anticoccidial drug replacement in broiler feed. Int. J. Vet. Sci. Med..

[2] Kim D.H., Yang H.M., Song J.Y., Park J., Kwon B.Y., Vu A.V., Lee D.S., Lee K.W. (2024). Effects of dietary mangosteen peel powder and extract on the growth performance, meat quality and indicators for immunity, gut health and antioxidant activity in broiler chicks. Poult. Sci..

[3] Liu Z.P., Chao J.R., Xu P.T., Lv H.Y., Ding B.Y., Zhang Z.F., Li L.L., Guo S.S. (2023). *Lonicera flos* and *Cnicus japonicus* extracts improved egg quality partly by modulating antioxidant status, inflammatory-related cytokines and shell matrix protein expression of oviduct in laying hens. Poult. Sci..

[4] Cheng X., Ning Z. (2023). Research progress on bird eggshell quality defects: A review. Poult. Sci..

[5] Benavides-Reyes C., Folegatti E., Dominguez-Gasca N., Litta G., Sanchez-Rodriguez E., Rodriguez-Navarro A.B., Umar Faruk M. (2021). Research Note: Changes in eggshell quality and microstructure related to hen age during a production cycle. Poult. Sci..

[6] Zhan X.-Z., Luo P., Zhang C., Zhang L.-J., Shen X., Jiang D.-L., Liu W.-J. (2024). Age-related changes in the mitochondrial, synthesis of steroids, and cellular homeostasis of the chicken ovary. Anim. Reprod. Sci..

[7] Fu Y., Zhou J., Schroyen M., Zhang H., Wu S., Qi G., Wang J. (2024). Decreased eggshell strength caused by impairment of uterine calcium transport coincide with higher bone minerals and quality in aged laying hens. J. Anim. Sci. Biotechnol..

[8] Hu Z., Wu L., Lv Y., Ge C., Luo X., Zhan S., Huang W., Shen X., Yu D., Liu B. (2025). Integrated analysis of microbiome and transcriptome reveals the mechanisms underlying the chlorogenic acid-mediated attenuation of oxidative stress and systemic inflammatory responses via gut-liver axis in post-peaking laying hens. J. Anim. Sci. Biotechnol..

[9] Feng J., Lu M., Ma L., Zhang H., Wu S., Qiu K., Min Y., Qi G., Wang J. (2023). Uterine inflammation status modulates eggshell mineralization via calcium transport and matrix protein synthesis in laying hens. Anim. Nutr. (Zhongguo Xu Mu Shou Yi Xue Hui).

[10] Shao L., Yan Y., Wang N., Tan Q., Huang Y., Lei L., Yang D., Liu L. (2023). Betulonic acid regulates oviduct epithelial cell inflammation through the TLR4, MAPK, and JAK/STAT signalling pathways. Reprod. Fertil. Dev..

[11] Somasundaram S., Sadique J. (1986). The role of mitochondrial calcium transport during inflammation and the effect of anti-inflammatory drugs. Biochem. Med. Metab. Biol..

[12] Kim H.R., Ryu C., Lee S.D., Cho J.H., Kang H. (2024). Effects of Heat Stress on the Laying Performance, Egg Quality, and Physiological Response of Laying Hens. Animals.

[13] Biswas S., Kim I.H. (2025). A thorough review of phytogenic feed additives in non-ruminant nutrition: Production, gut health, and environmental concerns. J. Anim. Sci. Technol..

[14] Movahedi F., Nirmal N., Wang P., Jin H., Grøndahl L., Li L. (2024). Recent advances in essential oils and their nanoformulations for poultry feed. J. Anim. Sci. Biotechnol..

[15] Feng J., Lu M., Wang J., Zhang H., Qiu K., Qi G., Wu S. (2021). Dietary oregano essential oil supplementation improves intestinal functions and alters gut microbiota in late-phase laying hens. J. Anim. Sci. Biotechnol..

[16] Youssef I.M., Aldhalmi A.K., Felemban S.G., Elsherbeni A.I., Khalil H.A., Hassan M.S., Abd El Halim H.S., Abd El-Hack M.E., Youssef K.M., Swelum A.A. (2024). Mannan oligosaccharides as a prebiotic for laying hens: Effects on fertility, hatchability, productive performance, and immunity. Transl. Anim. Sci..

[17] Majdalawieh A.F., Khatib B.K., Terro T.M. (2025). α-Mangostin Is a Xanthone Derivative from Mangosteen with Potent Immunomodulatory and Anti-Inflammatory Properties. Biomolecules.

[18] Kim H.J., Park S., Shin H.Y., Nam Y.R., Lam Hong P.T., Chin Y.W., Nam J.H., Kim W.K. (2021). Inhibitory effects of α-Mangostin on T cell cytokine secretion via ORAI1 calcium channel and K(+) channels inhibition. PeerJ.

[19] Dang T.H., Kim J.Y., Kim H.J., Kim B.J., Kim W.K., Nam J.H. (2023). Alpha-Mangostin: A Potent Inhibitor of TRPV3 and Pro-Inflammatory Cytokine Secretion in Keratinocytes. Int. J. Mol. Sci..

[20] Jonchère V., Réhault-Godbert S., Hennequet-Antier C., Cabau C., Sibut V., Cogburn L.A., Nys Y., Gautron J. (2010). Gene expression profiling to identify eggshell proteins involved in physical defense of the chicken egg. BMC Genom..

[21] Chen G., Li Y., Wang W., Deng L. (2018). Bioactivity and pharmacological properties of α-mangostin from the mangosteen fruit: A review. Expert Opin. Ther. Pat..

[22] Sultan O.S., Kantilal H.K., Khoo S.P., Davamani A.F., Eusufzai S.Z., Rashid F., Jamayet N.B., Soh J.A., Tan Y.Y., Alam M.K. (2022). The Potential of α-Mangostin from *Garcinia mangostana* as an Effective Antimicrobial Agent—A Systematic Review and Meta-Analysis. Antibiotics.

[23] Jian Z., Jinzhi S.U.N., Yuan Y.U.E., Bingkun Z. (2025). Research Progress on Biological Functions of α-Mangostin and Its Application in Broiler Production. Chin. J. Anim. Nutr..

[24] Pasaribu T., Sinurat A.P., Silalahi M., Lase J.A. (2024). Phytogenic cocktails fed in different feeding regimes as alternatives to antibiotics for improving performance, intestinal microbial, and carcass characteristics of slow growth chickens. Vet. World.

[25] Herawati O., Untari T., Anggita M., Artanto S. (2019). Effect of mangosteen (*Garcinia mangostana* L.) peel extract as an antibiotic growth promoter on growth performance and antibiotic resistance in broilers. Vet. World.

[26] Reyer H., Oster M., Zentek J., Männer K., Trakooljul N., Aumiller T., Wimmers K. (2026). Digestibility and transcriptomic profiling of kidney, pancreas, follicle, and uterus in laying hens supplemented with phytogenic feed additive. Poult. Sci..

[27] National Research Council (NRC) (1994). Nutrient Requirements of Poultry: Ninth Revised Edition 1994.

[28] Amireche D., Arzour-Lakehal N., Asma Nour el Houda B. (2025). Age-related variation and multivariate analysis of internal and external egg quality traits in ISA Brown hens. Rev. La Fac. Agron. Univ. Del Zulia.

[29] Hanusova E., Hrnčár C., Hanus A., Oravcova M. (2015). Effect of breed on some parameters of egg quality in laying hens. Acta Fytotech. Zootech..

[30] Zhou Q., Lan F., Gu S., Li G., Wu G., Yan Y., Li X., Jin J., Wen C., Sun C. (2023). Genetic and microbiome analysis of feed efficiency in laying hens. Poult. Sci..

[31] Molnár A., Maertens L., Ampe B., Buyse J., Kempen I., Zoons J., Delezie E. (2016). Changes in egg quality traits during the last phase of production: Is there potential for an extended laying cycle?. Br. Poult. Sci..

[32] Hu Z., Xu H., Zhang Z., Lu Y., Zhou Y., Zhu J., Deng Q., Wang X., Liu Y., Zhang Y. (2025). Comparative analysis of the performance, egg quality and ovarian immune function of fast and slow feather strains in tianfu green shell laying hens at various stages of egg production. Poult. Sci..

[33] Liu X., Shi L., Hao E., Chen X., Liu Z., Chen Y., Wang D., Huang C., Ai J., Wu M. (2024). Effects of 28 h ahemeral light cycle on production performance, egg quality, blood parameters, and uterine characteristics of hens during the late laying period. Poult. Sci..

[34] Zhu J., Liu Q., Wang Y., Zhu K., Guo J., Jin Y., Liu Y. (2024). Mangosteen extract reduces the bacterial load of eggshell and improves egg quality. Heliyon.

[35] Huang W., Lv Y., Zou C., Ge C., Zhan S., Shen X., Wu L., Wang X., Yuan H., Lin G. (2025). Mangosteen Pericarp Extract Mitigates Diquat-Induced Hepatic Oxidative Stress by NRF2/HO-1 Activation, Intestinal Barrier Integrity Restoration, and Gut Microbiota Modulation. Antioxidants.

[36] Fu T., Wang S., Liu J., Cai E., Li H., Li P., Zhao Y. (2018). Protective effects of α-mangostin against acetaminophen-induced acute liver injury in mice. Eur. J. Pharmacol..

[37] Wang H., Pan L., Si L., Ji R., Cao Y. (2021). Effects of Nrf2-Keap1 signaling pathway on antioxidant defense system and oxidative damage in the clams Ruditapes philippinarum exposure to PAHs. Environ. Sci. Pollut. Res. Int..

[38] Pedraza-Chaverri J., Cárdenas-Rodríguez N., Orozco-Ibarra M., Pérez-Rojas J.M. (2008). Medicinal properties of mangosteen (*Garcinia mangostana*). Food Chem. Toxicol..

[39] Tsai S.Y., Chung P.C., Owaga E.E., Tsai I.J., Wang P.Y., Tsai J.I., Yeh T.S., Hsieh R.H. (2016). Alpha-mangostin from mangosteen (*Garcinia mangostana* Linn.) pericarp extract reduces high fat-diet induced hepatic steatosis in rats by regulating mitochondria function and apoptosis. Nutr. Metab..

[40] Wang H., Pan L., Xu R., Si L., Zhang X. (2019). The molecular mechanism of Nrf2-Keap1 signaling pathway in the antioxidant defense response induced by BaP in the scallop Chlamys farreri. Fish Shellfish Immunol..

[41] John O.D., Mushunje A.T., Surugau N., Guad R.M. (2022). The metabolic and molecular mechanisms of α-mangostin in cardiometabolic disorders (Review). Int. J. Mol. Med..

[42] Li D., Liu Q., Lu X., Li Z., Wang C., Leung C.H., Wang Y., Peng C., Lin L. (2019). α-Mangostin remodels visceral adipose tissue inflammation to ameliorate age-related metabolic disorders in mice. Aging.

[43] Zuo J., Yin Q., Wang Y.W., Li Y., Lu L.M., Xiao Z.G., Wang G.D., Luan J.J. (2018). Inhibition of NF-κB pathway in fibroblast-like synoviocytes by α-mangostin implicated in protective effects on joints in rats suffering from adjuvant-induced arthritis. Int. Immunopharmacol..

[44] Gutierrez-Orozco F., Failla M.L. (2013). Biological activities and bioavailability of mangosteen xanthones: A critical review of the current evidence. Nutrients.

[45] Mohan S., Syam S., Abdelwahab S.I., Thangavel N. (2018). An anti-inflammatory molecular mechanism of action of α-mangostin, the major xanthone from the pericarp of *Garcinia mangostana*: An in silico, in vitro and in vivo approach. Food Funct..

[46] Bumrungpert A., Kalpravidh R.W., Chuang C.-C., Overman A., Martinez K., Kennedy A., McIntosh M. (2010). Xanthones from Mangosteen Inhibit Inflammation in Human Macrophages and in Human Adipocytes Exposed to Macrophage-Conditioned Media 1, 2. J. Nutr..

[47] Athanasiadou D., Jiang W., Goldbaum D., Saleem A., Basu K., Pacella M.S., Böhm C.F., Chromik R.R., Hincke M.T., Rodríguez-Navarro A.B. (2018). Nanostructure, osteopontin, and mechanical properties of calcitic avian eggshell. Sci. Adv..

[48] Jonchère V., Brionne A., Gautron J., Nys Y. (2012). Identification of uterine ion transporters for mineralisation precursors of the avian eggshell. BMC Physiol..

[49] Sah N., Mishra B. (2018). Regulation of egg formation in the oviduct of laying hen. World’s Poult. Sci. J..

[50] Hincke M.T., Gautron J., Tsang C.P.W., McKee M.D., Nys Y. (1999). Molecular Cloning and Ultrastructural Localization of the Core Protein of an Eggshell Matrix Proteoglycan, Ovocleidin-116*. J. Biol. Chem..

[51] Marie P., Labas V., Brionne A., Harichaux G., Hennequet-Antier C., Rodriguez-Navarro A.B., Nys Y., Gautron J. (2015). Quantitative proteomics provides new insights into chicken eggshell matrix protein functions during the primary events of mineralisation and the active calcification phase. J. Proteom..

[52] Gautron J., Stapane L., Le Roy N., Nys Y., Rodriguez-Navarro A.B., Hincke M.T. (2021). Correction to: Avian eggshell biomineralization: An update on its structure, mineralogy and protein tool kit. BMC Mol. Cell Biol..

[53] Bain M.M., Nys Y., Dunn I.C. (2016). Increasing persistency in lay and stabilising egg quality in longer laying cycles. What are the challenges?. Br. Poult. Sci..

[54] Rayan G., Fathi M. (2026). Ultrastructural changes in eggshell during incubation: Mechanisms and implications. Poult. Sci..

[55] Hamilton R.M.G., Bryden W.L. (2021). Relationship between egg shell breakage and laying hen housing systems—An overview. World’s Poult. Sci. J..

[56] van der Klein S.A.S., Zuidhof M.J., Bédécarrats G.Y. (2020). Diurnal and seasonal dynamics affecting egg production in meat chickens: A review of mechanisms associated with reproductive dysregulation. Anim. Reprod. Sci..

