# Supplementary material for: Effects of Dietary Supplementation with α-Mangostin on Oviduct Inflammation and Eggshell Quality in Aging Laying Hens
Source: Animals (Basel). 2026 Apr 5;16(7):1118. doi: 10.3390/ani16071118 (PMC13072074; doi:10.3390/ani16071118)
Supplement: Supplementary file 1 [file animals-16-01118-s001.zip › Supplementary Table S1..pdf]

Supplementary Table S1

Primer	5' to 3'
SLC4A1-F	CGTCGATGGGTGGATGAGGG
SLC4A1-R	AGGGCACAACTGAACAGCAC
SLC4A2-F	TACCCCCATTACCTGAGCGA
SLC4A2-R	GCGCGGCAAAGTAGATGAAG
SLC4A7-F	AAGTGGCCCCTGAGGTATCC
SLC4A7-R	CCTGTGCTTCTCCATCAGGT
SLC4A9-F	TGGAGAAACTCCCAAGCACAG
SLC4A9-R	GGCATGTCCTCAGCTCAAAG
ATP6V0D2-F	CAGCGACCCATAGCTGAACT
ATP6V0D2-R	AGCTAATGGCGTGTCAACCA
ATP6V1C2-F	AAGAGTTGGAGACTGCTGCG
ATP6V1C2-R	GCAAATCCACGTGTGACAGC
ATP6V1G3-F	AACGTAATGGGCTCCCAAGG
ATP6V1G3-R	CTGCTGGTGAGGTTTCGGAT
TRPV6-F	CTGTGCTCACGTCCTCTGTT
TRPV6-R	TACGTGACAGATGGTGGCAG
ATP2B2-F	ACACAAACACTGCATCACGC
ATP2B2-R	TCCCATTATGCAGCACCCAG
CA2-F	TACGACAGCCACAACGGAC
CA2-R	ACTCCACGTTGAAGGAGTGC
LYZ（Lysozyme）-F	TGAAGCGTCACGGACTTGAT
LYZ（Lysozyme）-R	CTCCCATCGGTGTTACGGTT
EDIL3-F	ATGGTGGCATCTGTCTGTCG
EDIL3-R	CAGGGTCCTGCTGAAGTTGG
MFGE8-F	GTACATCCGCATCTACCCCG
MFGE8-R	AGCAACCTGCCGTGTTGAAA
OC-17-F	GAGGAGGCCTTCACCTCGT
OC-17-R	TGGGTCCGTTTATTGCAGTGT
OC-116(MEPE)-F	CAGCTCCCAGCAAAGCCTAT
OC-116(MEPE)-R	GTGCTCCATCTCTGTCAGGC
GAPDH-F	GTCAAGGCTGAGAACGGGAA
GAPDH-R	GCCCATTTGATGTTGCTGGG
